# Supplementary material for: Maladaptive Compensatory Neural Mechanisms Associated with Activity-Related Osteoarthritis Pain: Dissociation of Psychological and Activity-Related Neural Mechanisms of WOMAC Pain and VAS Pain
Source: J Clin Med. 2025 May 22;14(11):3633. doi: 10.3390/jcm14113633 (PMC12155135; doi:10.3390/jcm14113633)
Supplement: Supplementary file 1 [file jcm-14-03633-s001.zip › jcm-3443652-supplementary.pdf]

# Maladaptive compensatory neural mechanisms associated with activity-related osteoarthritis pain: dissociation of the neural mechanisms of WOMAC pain and VAS pain

Marta Imamura[1], Kevin Pacheco-Barrios[2,3], Paulo Sampaio de Melo[2], Anna Marduy[2], Linamara Battistella[1], Marcel Simis[1], Felipe Fregni[2]

## Supplementary material S1

### Functional Clinical Assessment Description and Application

*Adapted from our protocol under review – Deficit of inhibition as a marker of neuroplasticity - DEFINE study - in rehabilitation: a longitudinal cohort study protocol - Sims et al. 2021 [1]

Montreal Cognitive Assessment (MOCA): Evaluates the patient's abilities regarding their cognitive functions. Assesses eight cognitive function domains including: executive function, visuo-spatial ability, memory, attention, concentration, occupational memory, language, and temporal and spatial orientation. This assessment takes approximately 10 minutes to be applied and reaches a maximum score of 30 points. Lower scores indicate higher cognitive impairment.[2]

Pain Catastrophizing Scale: This scale consists of nine components judged through a Linkert scale of 5 items varying from "almost never" to "almost always" in the extremities. The scale is scored by adding all the components and dividing them by the number of answered items. The

minimum score is 0 and the maximum 5, with higher scores indicating higher levels of catastrophizing thoughts. [3, 4]

Visual Analogue Scale (VAS) for Pain: This is a linear scale consisting in a 10cm line jotted on a piece of paper. Its extremities indicate its linearity character by depicting "no pain" on one of the ends and "maximum pain" on the other. Individuals are asked to identify their level of discomfort on the line. The subject's pain level is judged by how close their mark is to the extremities: marks closer to 0cm ("no pain" extremity) indicate lower levels of pain and marks closer to the 10cm end ("maximum pain") indicate higher levels of pain. Subjects were asked to identify their pain in the last 48h by making a perpendicular mark to the VAS line. [5]

6-minute and 10 meters gait test:[6]

10-meter gait test: evaluates a subject's speed for a short-distance walk. Individuals are asked to walk at their normal speed and are recommended to walk 14 meters total so that the initial and final 2 meters are discounted.

6-minute gait test: assess the maximum distance an individual can walk on a flat, rigid surface in six minutes. Subjects walk on a 30-meter track that laps every 3 meters with turning points identified with a cone.

Timed Up and Go (TUG): Used to assess a subject's mobility status. This test measures the amount of time it takes for subjects to stand up from a chair, walk three meters, turn around and sit back on the chair.[7]

Berg Balance Scale: this is a 14-item list scale designed to evaluate an individual's ability to stably balance during various tasks including reaching, turning, transferring, standing, and standing up. Each task is scored from 0-4 according to the subject's ability to maintain balance, with a maximum score of 56 points. Higher scores mean better balance. [8]

Epworth sleepiness scale: Assesses the level of daytime sleepiness. This is a self-applicable survey that presents 8 different daily scenarios and evaluates the probability f an individual falling asleep in these situations. [9]

Hamilton Depression Rating Scale (HAM-D): This is a 17-item scale that investigates the individual's mood within the last seven days, including the day of its application. The items are evaluated through a Likert scale that may range from 0 to 2 or 0 to 4 according to each item. The maximum score is 52, and depression is verified with a score of 8 points or higher.[10]

Hospital Anxiety and Depression Scale (HADS): This instrument consists of 14 multiple-choice questions that quantify symptoms of anxiety and depression. It is divided into two subscales for depression and anxiety separately, each made up of 7 items. Achievable scores for each subscale range from 0 to 21. The scale aims to identify mild levels of affective disorders outside of the psychiatric environment and evaluates the subject's mood during the last seven days. [11]

Western Ontario and McMaster Universities Osteoarthritis Index (WOMAC): This is a three-part scale evaluating pain, stiffness, and physical function of individuals with knee and hip

osteoarthritis. It is a 24-component questionnaire with 5 items inquiring about pain, 2 items about stiffness, and 17 items about physical function. Scores for each item range from 0 to 4 with higher scores indicating higher symptom levels or more disability. Scores can be presented for each subscale with a maximum of 20 points for the pain scale, 8 points for the stiffness scale, and 68 points for the physical function scale, but can also be presented as a total sum of all three domains.[12]

Kellgren-Lawrence Radiographic Classification of OA: This classification relates to disease severity in knee osteoarthritis. It is divided into five degrees ranging from 0 to 4 where 0 indicates no signs of osteoarthritis and 4 indicates large osteophyte formations with narrowing of joint space, deformity of bony extremities, and severe sclerosis.[13]

36-item short form (SF-36): This 36-item questionnaire encompasses eight different domains: 1) physical functioning (10 items); 2) Role limitations due physical problems (4 items); 3) social functioning (2 items); 4) bodily pain (2 items); 5) general mental health (5 items); 6) role limitations due emotional problems (3 items); 7) vitality (4 items); and 8) general health perceptions (5 items). Scores indicate health status in which a lower score correlates to lower health status, and a higher score indicates higher health status.[14]

**REFERENCES**

1. Simis M, Imamura M, de Melo P, Marduy A, Battistella L, Fregni F. Deficit of inhibition as a marker of neuroplasticity (DEFINE study) in rehabilitation: a longitudinal cohort study protocol. Frontiers in Neurology. 2021;12:1193.

2. Freitas S, Simões MR, Alves L, Santana I. Montreal Cognitive Assessment (MoCA): normative study for the Portuguese population. J Clin Exp Neuropsychol. 2011;33(9):989-96. Epub 2011/11/16. doi: 10.1080/13803395.2011.589374. PubMed PMID: 22082082.

3. Sullivan MJL, Bishop SR, Pivik J. The Pain Catastrophizing Scale: Development and validation. Psychological Assessment. 1995;7(4):524-32. doi: 10.1037/1040-3590.7.4.524.

4. Sardá Junior J, Nicholas MK, Pereira IA, Pimenta CADM, Asghari A, Cruz RM. Validation of the Pain-Related Catastrophizing Thoughts Scale. Acta Fisiátrica. 2008;15(1). doi: 10.5935/0104-7795.20080001.

5. Williamson A, Hoggart B. Pain: a review of three commonly used pain rating scales. J Clin Nurs. 2005;14(7):798-804. Epub 2005/07/08. doi: 10.1111/j.1365-2702.2005.01121.x. PubMed PMID: 16000093.

6. Steele B. Timed walking tests of exercise capacity in chronic cardiopulmonary illness. J Cardiopulm Rehabil. 1996;16(1):25-33. Epub 1996/01/01. doi: 10.1097/00008483-199601000-00003. PubMed PMID: 8907439.

7. Podsiadlo D, Richardson S. The timed "Up & Go": a test of basic functional mobility for frail elderly persons. J Am Geriatr Soc. 1991;39(2):142-8. Epub 1991/02/01. doi: 10.1111/j.1532-5415.1991.tb01616.x. PubMed PMID: 1991946.

8. Stevenson TJ. Detecting change in patients with stroke using the Berg Balance Scale. Aust J Physiother. 2001;47(1):29-38. Epub 2001/09/13. doi: 10.1016/s0004-9514(14)60296-8. PubMed PMID: 11552860.

9. Johns MW. Reliability and factor analysis of the Epworth Sleepiness Scale. Sleep. 1992;15(4):376-81. Epub 1992/08/01. doi: 10.1093/sleep/15.4.376. PubMed PMID: 1519015.

10. Freire MÁ, Figueiredo VLMD, Gomide A, Jansen K, Silva RAD, Magalhães PVDS, et al. Escala Hamilton: estudo das características psicométricas em uma amostra do sul do Brasil. Jornal Brasileiro de Psiquiatria. 2014;63(4):281-9. doi: 10.1590/0047-2085000000036.

11. Botega NJ, Bio MR, Zomignani MA, Garcia C, Jr., Pereira WA. [Mood disorders among inpatients in ambulatory and validation of the anxiety and depression scale HAD]. Rev Saude Publica. 1995;29(5):355-63. Epub 1995/10/01. doi: 10.1590/s0034-89101995000500004. PubMed PMID: 8731275.

12. McConnell S, Kolopack P, Davis AM. The Western Ontario and McMaster Universities Osteoarthritis Index (WOMAC): a review of its utility and measurement properties. Arthritis & Rheumatism. 2001;45(5):453-61. doi: 10.1002/1529-0131(200110)45:5<453::aid-art365>3.0.co;2-w.

13. Kellgren JH, Lawrence JS. Radiological assessment of osteo-arthrosis. Ann Rheum Dis. 1957;16(4):494-502. Epub 1957/12/01. doi: 10.1136/ard.16.4.494. PubMed PMID: 13498604; PubMed Central PMCID: PMCPMC1006995.

14. Ware JE, Jr., Sherbourne CD. The MOS 36-item short-form health survey (SF-36). I. Conceptual framework and item selection. Med Care. 1992;30(6):473-83. Epub 1992/06/11. PubMed PMID: 1593914.

# Maladaptive compensatory neural mechanisms associated with activity-related osteoarthritis pain: dissociation of the neural mechanisms of WOMAC pain and VAS pain

Marta Imamura[1], Kevin Pacheco-Barrios[2,3], Paulo Sampaio de Melo[2], Anna Marduy[2], Linamara Battistella[1], Marcel Simis[1], Felipe Fregni[2]

**Supplementary material S2**

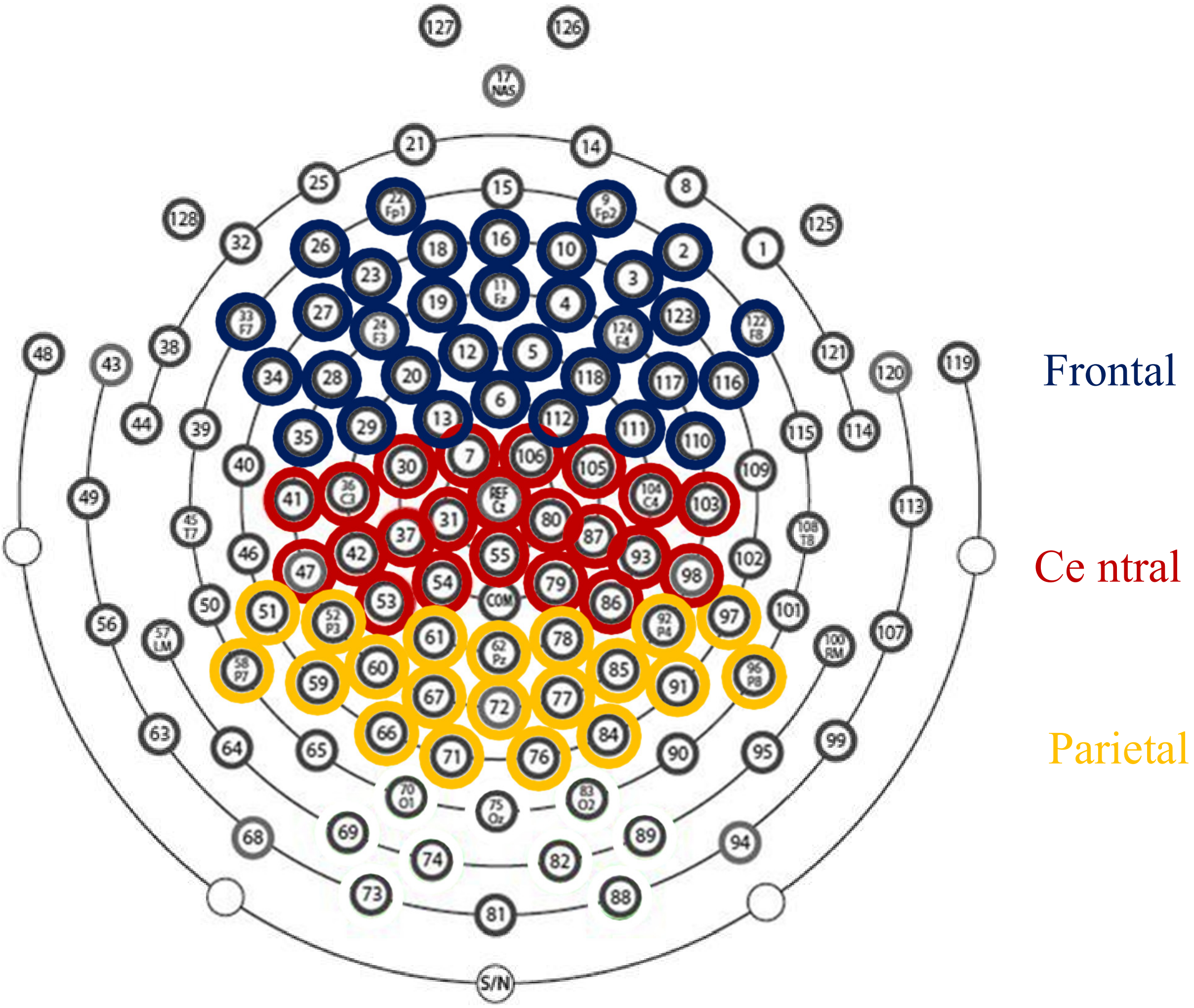


Maladaptive compensatory neural mechanisms associated with activity-related osteoarthritis pain: dissociation of the neural mechanisms of WOMAC pain and VAS pain

Marta Imamura[1], Kevin Pacheco-Barrios[2,3], Paulo Sampaio de Melo[2], Anna Marduy[2], Linamara Battistella[1], Marcel Simis[1], Felipe Fregni[2].

Supplementary material S3 – Univariate Analysis

| Variables | Beta-coefficient | 95% CI | p-value |
|---|---|---|---|
| **WOMAC pain** | | | |
| Body mass index | 0.153 | 0.013 to 0.294 | 0.033 |
| WOMAC total | 0.189 | 0.171 to 0.209 | < 0.01 |
| Pain catastrophizing | 0.178 | 0.118 to 0.237 | < 0.01 |
| Hospital Anxiety and Depression scale - Anxiety | 0.439 | 0.279 to 0.599 | < 0.01 |
| Hamilton Depression scale | 0.356 | 0.235 to 0.476 | < 0.01 |
| Education | -1.372 | -2.297 to -0.446 | 0.004 |
| MOCA | -0.325 | -0.474 to -0.348 | < 0.01 |
| Epworth sleepiness scale | 0.221 | 0.094 to 0.334 | 0.001 |
| K-L classification | 0.676 | 0.005 to 1.347 | 0.048 |
| 6-minutes-walking test | -0.014 | -0.020 to -0.007 | < 0.01 |
| SF-36 | -0.124 | -0.154 to -0.095 | < 0.01 |
| *QST* | | | |
| Pain threshold superior limb | -0.517 | -0.886 to -0.148 | 0.006 |
| Pain threshold knee | -0.545 | -0.833 to -0.257 | < 0.01 |
| CPM | -0.969 | -1.614 to -0.325 | 0.004 |
| **VAS pain** | | | |
| WOMAC total | 0.062 | 0.045 to 0.079 | < 0.01 |
| Pain catastrophizing | 0.072 | 0.038 to 0.106 | < 0.01 |
| Hamilton Depression scale | 0.105 | 0.035 to 0.174 | 0.003 |
| Education | -0.879 | -1.316 to -0.443 | < 0.01 |
| MOCA | -0.161 | -0.235 to -0.087 | < 0.01 |
| Epworth sleepiness scale | 0.078 | 0.011 to 0.144 | 0.022 |
| K-L classification | 0.608 | 0.276 to 0.941 | < 0.01 |
| 6-minutes-walking test | -0.008 | -0.011 to -0.005 | < 0.01 |
| 10-meters-walking test | 0.107 | 0.054 to 0.160 | < 0.01 |
| Time up-and-go | 0.074 | 0.026 to 0.122 | 0.003 |
| Berg Balance scale | -0.104 | -0.147 to -0.060 | < 0.01 |
| SF-36 | -0.044 | -0.063 to 0.026 | < 0.01 |
| *QST* | | | |
| Pain threshold superior limb | -0.246 | -0.437 to -0.055 | 0.012 |
| Pain threshold knee | -0.211 | -0.365 to -0.057 | 0.008 |
